# Brands, costs and registration status of antimalarial drugs in the Kenyan retail sector

**DOI:** 10.1186/1475-2875-4-36

**Published:** 2005-07-26

**Authors:** Abdinasir A Amin, Robert W Snow

**Affiliations:** 1 Malaria Public Health & Epidemiology Group, Centre for Geographic Medicine, Kenya Medical Research Institute/Wellcome Trust Collaborative Programme, P.O. Box 43640, Nairobi, 00100 GPO, Kenya; 2 Centre for Tropical Medicine, University of Oxford, John Radcliffe Hospital, Headington, Oxford, OX3 9DU, UK

## Abstract

**Background:**

Although an important source of treatment for fevers, little is known about the structure of the retail sector in Africa with regard to antimalarial drugs. This study aimed to assess the range, costs, sources and registration of antimalarial drugs in the Kenyan retail sector.

**Methods:**

In 2002, antimalarial drug registration and trade prices were established by triangulating national registration lists, government gazettes and trade price indices. Data on registration status and trade prices were compared with similar data generated through a retail audit undertaken among 880 randomly sampled retailers in four districts of Kenya.

**Results:**

Two hundred and eighteen antimalarial drugs were in circulation in Kenya in 2002. These included 65 "sulfur"-pyrimethamine (sulfadoxine-pyrimethamine and sulfalene-pyrimethamine (SP), the first-line recommended drug in 2002) and 33 amodiaquine (AQ, the second-line recommended drug) preparations. Only half of SP and AQ products were registered with the Pharmacy and Poisons Board. Of SP and AQ brands at district level, 40% and 44% were officially within legal registration requirements. 29% of retailers at district level stocked SP and 95% stocked AQ. The retail price of adult doses of SP and AQ were on average 0.38 and 0.76 US dollars, 100% and 347% higher than trade prices from manufacturers and importers. Artemether-lumefantrine, the newly announced first-line recommended antimalarial drug in 2004, was found in less than 1% of all retail outlets at a median cost of 7.6 US dollars.

**Conclusion:**

There is a need to ensure that all antimalarial drugs are registered with the Pharmacy and Poisons Board to facilitate a more stringent post-marketing surveillance system to ensure drugs are safe and of good quality post-registration.

## Background

Drugs' retailers play an important role in the management of childhood fevers in Africa [1,2]. The extent of self-medication with proprietary drugs from retailers varies across the continent and has been reported to be as low as 19% in Guinea to as high as 94% in some parts of rural Ghana [3]. Reasons for reported use of the retail sector for fever management are diverse, ranging from ease of geographical access [4] to economic accessibility [5] and perceived failures of the formal health sector [3,6].

The World Health Organization (WHO) has recognised the role of the retail sector in helping to meet international targets on prompt access to antimalarial drugs [2,7]. Despite the renewed interest in home-based care, there is still a relatively poor understanding of the structure of the retail sector compared to our knowledge of formal service providers. While there has been a plethora of research on the use of the retail sector by communities in malaria endemic areas of Africa [8], complimented by a few studies on the knowledge of antimalarial drugs by service providers in the retail sector [9-12], to-date there has been only one study that has characterized the structure of the retail sector with respect to antimalarial drug ranges and sources, and how these relate to the national supply [13].

In Kenya, the retail sector is an important source of treatment for fevers [9,14-19] and the aim of this paper is to characterize the antimalarial drug products provided by this sector in terms of legal status, product ranges, costs and sources. The results focus on first and second-line treatment for uncomplicated malaria at the time of the studies, "sulfur"-pyrimethamine (sulfadoxine-pyrimethamine and sulfalene-pyrimethamine, SP) and amodiaquine (AQ), respectively. Issues related to the availability of other antimalarial products, including those that are no longer promoted as efficacious (chloroquine) and those that will serve as replacement therapies for SP and AQ in 2005 (the artemisinins) are highlighted.

## Methods

### National antimalarial audit

A national list of all registered antimalarial drugs was obtained from the Pharmacy and Poisons Board (PPB) of the Ministry of Health (MoH) [20,21] and updated through a series of reviews of minutes of the PPB and Kenya Gazette notices. Gazette notices are provided by the Government Printers and notify the public of registration of new products. The Committee for Drug Registration (CDR) of the PPB registers drugs based on their safety, quality and efficacy and unregistered drugs are considered illegal. Applicants pay 500 US dollars for Kenyan products and 1,000 US dollars for imported products per application. Registration is valid for five years, after which a re-registration is sought from the CDR for a further five years with a second fee of 300 US dollars and 500 US dollars for local and imported products respectively.

The official PPB list was augmented by two commercially available drugs and medical devices price lists [22,23], published yearly or bi-monthly, and serving as national trade indices. These lists provided information not available from the PPB sources. Information collected from all sources included brand names, dosage forms, strengths, manufacturers, trade packs, trade costs and product registration status and registration dates. The composite national antimalarial database was finalized on May 31, 2002 to enable comparison with subsequent retail audits at district level.

### Retail audit survey procedures

A retail audit was undertaken between February and June 2002 in four malaria monitoring sentinel districts: Kwale in Coast Province, Makueni in Eastern Province, Greater Kisii in the western highlands and Bondo on the shores of Lake Victoria. These are described elsewhere in detail [4,19,24].

A national census of retailers was developed between 1999 and 2000 by a commercial market research organisation. Data for each of the four sentinel districts were purchased and used to identify outlets that stocked and sold antimalarial or antipyretic products at the time of the retail census. Data were then displayed in MapInfo (Version 6.0, 1985–2000) and physical addresses compared against coordinates of market centres obtained from topographic maps and GPS data from various sources [4,25]. Any errors in positioning the 1999–2000 data were then corrected and positions of outlets redefined to the market centre. Outlets were categorized into pharmacies, large retailers (defined crudely as stores with more than one person serving customers during normal working hours), and small retailers (defined as outlets with only one person serving customers during normal working hours). Outlets were then sampled based upon the estimates of the numbers of retail outlets in each district and the expected prevalence of SP stocks (assumed to be 50%) to achieve between 5–10% precision and 95% confidence in the parameters of interest. A minimum of 20 pharmacies in each district was targeted, large retailers were randomly sampled to achieve a minimum of 40 outlets per district, and a random sample of 160 small retailers per district was selected.

Between February and May 2002, districts were visited to a) confirm which outlets in the sampling frame were retailing antimalarial drugs (the main drugs of interest); b) establish precise geographical positions of the outlet using a hand-held GPS unit (Magellan GPS 315 or Garmin etrex); and c) obtain permission from shopkeepers for a more indepth interview at a later date. In June 2002, a retail audit was undertaken among retailers who consented to the capture of information on brands of antimalarial drugs, pharmacological groups, wholesale source and retail costs. Data were entered twice using MS-Access 2000 (Microsoft Corp., Redmond, USA) developed data-entry screens, verified and cleaned. Data were analysed using a combination of MS Excel 2000 (Microsoft Corp., Redmond, USA) and SPSS version 9.0 for Windows (SPSS Inc., Chicago, USA) and presented as proportions, medians and interquartile ranges.

## Results

### Range of products at the national level

One hundred and thirty five oral antimalarial products registered with the PPB were identified. Of these, registration dates were gazetted for 122 (90.4%), the remaining 13 (9.6%) were noted in the minutes of the CDR as approved for registration, but no registration dates or reference numbers were available. Of the 122 products for which details were available, only 40 (32.8%) were within their five-year registration period, and 82 (67.2%) were due for re-registration. It is possible that some products in the latter group had been granted marketing approval by the PPB, but were awaiting final gazettement and registration (and could, therefore, not be found on official lists) or that the manufacturers and dealers no longer marketed these products and had, therefore, allowed registration to lapse. Of those with an expired registration status, however, 39 (47.6%) were identifiable on the commercial trade indices, suggesting they were still marketed in Kenya.

83 products were identified that were not on the PPB list, but were available on commercial lists. It is estimated, therefore, that 218 oral antimalarial products were in circulation in 2002. Of these products, 92 (42.2%) were manufactured locally and 126 (57.8%) were products imported from overseas.

Table 1 shows the overall registration status of the 218 products according to the various antimalarial classes. From the national audit, 34/65 (52.3%) of SP products were registered, while 17/33 (51.5%) of AQ products were registered with the PPB. All artemisinin (ART) tablets, mefloquine (MEF) tablets and halofantrine (HAL) products were registered, while none of the ART suspensions were registered.

**Table 1:** National and retail audit of oral anti-malarial drugs available on the Kenyan market in 2002

	**National audit**		**Retail audit**		
**Generic Name**	# Brands identified (registered)*	**Median (IQR) cost (USD) of treatment course**^†^	# Brands identified (registered)*	**# Outlets stocking (%)**	**Median (IQR) cost (USD) of treatment course**^†^

SP tablets	49 (29)	0.19 [0.13, 0.32]	30 (16)	250 (28.5%)	0.38 [0.25, 0.65]
SP suspensions and drops^‡^	16 (5)	0.39 [0.28, 0.53]	15 (2)	57 (6.5%)	0.44 [0.44, 0.56]
AQ tablets	22 (12)	0.17 [0.14, 0.55]	13 (6)	818 (93.4%)	0.76 [0.76, 0.76]
AQ suspensions	11 (5)	0.28 [0.15, 0.49]	12 (5)	71 (8.1%)	0.51 [0.39, 0.60]
CQ tablets	43 (33)	0.09 [0.08, 0.19]	12 (5)	132 (15.1%)	0.44 [0.25, 0.44]
CQ syrups	22 (10)	0.05 [0.03, 0.30]	9 (4)	12 (1.4%)	0.26 [0.06, 0.31]
QN tablets	25 (19)	2.73 [2.40, 3.16]	3 (2)	32 (3.7%)	3.20 [2.40, 4.00]
QN drops and mixtures	3 (1)	2.24 [1.87, 3.00]	4 (1)	49 (5.6%)	4.00 [3.27, 4.00]
ART tabs	11 (11)	5.34 [4.16, 5.56]	7 (7)	24 (2.7%)	7.11 [6.14, 7.96]
ART suspensions	1 (0)	3.86 [3.86, 3.86]	2 (0)	21 (2.4%)	5.00 [4.44, 5.13]
MEF tablets	4 (4)	5.04 [3.83, 9.53]	4 (4)	14 (1.6%)	7.61 [7.33, 7.87]
HAL tablets	1 (1)	7.96 [7.96, 7.96]	1 (1)	22 (2.5%)	9.90 [9.26, 10.25]
HAL suspensions	1 (1)	2.83 [2.83, 2.83]	1 (1)	18 (2.1%)	3.55 [3.20, 3.71]
Other tablets	9 (6)	3.62 [0.35, 13.20]	1 (1)	18 (2.1%)	17.51 [17.51, 17.51]

* Registration period covers up to and including May 31, 2002.

^† ^For packaged commodities, the calculations were derived per tablet and per recommended dose for adults. Where possible, large, bulk packaging was selected for individual suppliers to provide the cheapest values for the national audit.

^‡ ^Liquid dosage forms (suspensions, syrups, mixtures and paediatric drops) were all costed per dosage per child aged 1–5 years – not adult treatment courses. The mean dose per product was calculated as the mid-point between the Division of Malaria Control (DOMC) recommended dose for a 1 year old (lower limit of 10 kg) and a 5 year old (upper limit of 18 kg).

### Range of antimalarial drug classes and formulations at district level

Eight hundred and eighty retailers were sampled, but four were excluded from analysis since the shops remained closed even after three visits (Table 1). Overall, SP was stocked by 28.7% of retailers and AQ by 94.7%. Chloroquine (CQ), which had been replaced by SP in 1998 as the first-line recommended drug, was still available in 15.4% of retail outlets. Other antimalarial drugs were available in less than 10% of retail outlets. SP and AQ tablets were the most widely stocked formulations (28.5% and 93.4%, respectively) and were available in pharmacies, large retailers, and small retailers. ART, HAL and MEF were sold exclusively in pharmacies.

### Range, availability and registration of antimalarial brands at district level

Thirty brands of SP tablets were identified in the districts (Table 1), the two most widely stocked being Falcidin^® ^(stocked by 19.9% of outlets, Cosmos Limited, Kenya) and Fansidar^® ^(8.8%, L. Hoffmann La Roche, Switzerland). Sixteen SP brands (53.3%) were registered with the PPB. Zentakelfin^®^, Sudorin^® ^and Lansidar^®^, all unregistered, were available in some district level pharmacies and not recorded during the national audit. Fifteen brands of SP suspensions were also found, yet only two were registered with the PPB.

For AQ tablets, 13 brands were identified of which six (46.2%) were registered with the PPB. Three brands (which were not registered) were identified in the districts and were not recorded during the national audit: Amowin^®^, Vanida^®^, and Maratab^®^. Malaratab^® ^(Cosmos Limited, Kenya) was the most widely stocked AQ tablet, found in 87.9% of outlets. Twelve brands of AQ syrup were encountered with only five (41.7%) registered with the PPB.

Of interest was that all higher order antimalarial tablets, such as quinine (QN), HAL, MEF and ART class of drugs, were to a large extent all registered with the PPB and mostly available in pharmacies. The exception was one QN tablet formulation. Conversely, only 2/5 (40%) of the higher order antimalarial syrups were registered.

### Wholesale sources of antimalarial drugs to district level retailers

Due to the wide range of products in pharmacies, it was not possible to ask the wholesale source of individual products. Respondents were asked the primary wholesale source of antimalarial drugs in stock at the time. For large and small retailers, the source of each product audited was established. The wholesale source of most products in stock was adopted as the primary source of antimalarial drugs for each outlet. Drug sources thus defined were classified in eight groups as shown in Table 2. Results show that overall pharmacies obtained their antimalarial drugs from pharmaceutical wholesalers outside the districts (67.1%). Large retailers obtained their drugs from general wholesalers outside the districts (39.8%) or inside the district (34.8%). Most small retailers (45.3%) obtained antimalarial drugs from general wholesalers within the districts. A substantial proportion of small retailers (23.3%) also obtained their antimalarial drugs from general wholesalers outside the districts. Mobile vendors supplied a good number of small retailers (14.0%) and large retailers (7.5%), but not pharmacies.

**Table 2:** Primary wholesale sources of antimalarial products to 876* retailers in the four study districts.

	**Totals**		
	**Pharmacies**	**Large Shops**	**Small Shops**

Mobile vendors	0	12 (7.5%)	90 (14.0%)
General wholesalers-within district	0	56 (34.8%)	292 (45.3%)
General wholesaler-outside district	2 (2.9%)	64 (39.8%)	150 (23.3%)
Pharmaceutical wholesaler-within district	17 (24.3%)	11 (6.8%)	51 (7.9%)
Pharmaceutical wholesaler-outside district	47 (67.1%)	3 (1.9%)	13 (2.0%)
Pharmaceutical company	2 (2.9%)	3 (1.9%)	0
Drug representative	0	7 (4.3%)	5 (0.8%)
Unknown	2 (2.9%)	5 (3.1%)	44 (6.8%)

* Four small retailers were excluded from analysis since they remained closed even after three repeated visits.

### Trade versus retail costs of antimalarial drugs

For product costs, standardized dose regimens were used to enable comparisons between the antimalarial classes. SP, AQ, CQ and QN doses were based on the malaria standard treatment guidelines set by the DOMC of the MoH [26]. For all other antimalarial drugs (which are not the subject of DOMC guidelines), the East African Pharmaceutical Loci, a regional formulary for healthcare professionals, was used [22]. Costs were calculated in US dollars (USD) based on the Central Bank of Kenya rates at the time of the survey (June 2002).

For trade prices, large bulk packaging was selected per supplier to provide the cheapest factory gate costs (Table 1). The trade price for an adult dose of SP was 0.19 USD (IQR 0.13, 0.32), while a standard dose for a paediatric patient on SP suspensions cost nearly twice as much. AQ tablet and suspension prices were 0.17 USD for an adult dose and 0.28 USD for a paediatric suspension. The trade price range for QN, MEF, HAL and ART products was between 2.24 and 7.96 USD (Table 1).

Retail prices at the district level were standardized to an adult and paediatric dose as described above and are shown in Table 1. The retail price for an adult dose of SP was 0.38 USD (IQR 0.25, 0.65), while a standard dose for a paediatric patient on SP suspension was 0.44 USD (IQR 0.44, 0.56). AQ tablet prices were 0.76 USD (IQR 0.76, 0.76), while AQ suspension cost a median of 0.51 USD (0.39, 0.60). The retail price range for QN, MEF, HAL and ART products was between 3.55 to 9.90 USD (Table 1).

The two most widely stocked brands of SP and AQ tablets (Falcidin^® ^and Malaratab^®^, respectively) are used to demonstrate retail mark-ups on trade costs. The trade cost for an adult treatment course of Falcidin^® ^in May 2002 was 0.15 USD, while that for Malaratab^® ^was 0.55 USD. The equivalent median retail prices for the two products were 0.32 USD and 0.76 USD, respectively, representing a 113% and 38% mark-up on the trade price.

## Discussion

There was a wide range of antimalarial products available in the Kenyan retail market in 2002. However, not all nationally marketed first- and second-line drugs were in circulation in the peripheral retail sector; 17 preparations of SP (26%) and five (15%) of AQ were not detected at the district level. Conversely, there were three brands of SP and three of AQ which were in retail circulation at the district level, but which had not been identified during the central audit and were unregistered by the PPB. One serious consequence of a wide range of products available to largely biomedically ill-informed, rural populations is brand confusion, which may lead to unintentional repeated doses of the same drug class and consequently, dose-dependent adverse effects [27]. Moreover, the availability of unregistered products poses a danger to malaria treatment since the safety, efficacy and quality of such drugs cannot be guaranteed [28].

The most widely available antimalarial in the retail sector was AQ, sold in 95% of outlets surveyed. AQ was a prescription-only medication (POM) and regarded as the second-line treatment at the time of the survey. In contrast, SP, the first-line drug in 2002, was available in only 29% of outlets surveyed. The situation was found to be different in neighbouring Tanzania when CQ was still the first-line drug for uncomplicated malaria. 33% of general retailers and 98% of pharmacies stocked CQ. Conversely SP, the second-line treatment, was sold in less than1% of general retailers and in 37% of pharmacies [13]. Although the apparent policy-practice disconnect in Kenya in drug scheduling and national malaria policy could be attributed to a more prolific and unregulated retail market in antimalarial drugs compared to Tanzania, a more plausible explanation is a lack of a concerted national effort to inform populations about changes in drug policy. In the absence of strong government action to back up new drug policies (e.g. mass communication, training of health workers, etc.), market forces will fill the gap and dictate the stocking of antimalarial drugs by retailers. Closer cooperation and consultation with local pharmaceutical manufacturers and importers of antimalarial drugs during national policy change and broad, high profile, community-wide communication strategies for drug policy change are critical for successful implementation.

Compared to the national trade price data, mark-ups are between 100–347% (using SP and AQ tablets as examples) when they reach the peripheral retail level. Retailers represent the last link in a chain of manufacturers/drug importers and wholesalers, and it is not clear from the results if the high price mark-ups on antimalarial drugs are transferred from these primary/middle level suppliers to the retailer or these represent retailer pricing structures. More formative research is required to understand the pricing structure for antimalarial drugs in the retail sector to identify opportunities to reduce costs and retail profit margins with new medicines such as artemisinin-based combination therapies (ACTs).

At the time of this study, the artemisinins (potential replacements to SP monotherapy) were only available in pharmacies in Kenya. More importantly, the proposed new first-line drug for Kenya, artemether-lumefantrine (registered in Kenya in July 1999 as Coartem^®^), was available in 8/70 (11%) of district level pharmacies (i.e. less than 1% of all retailers) at a median cost of 7.6 US dollars (interquartile range 6.2, 10.1). This represents a huge cost to households which invariably bear most of the cost of treating malaria [29].

## Conclusion

The studies show that when drug classes become established treatments, many branded options soon become available on the retail market. In Kenya, the regulation of this plethora of brands is weak: many do not go through the regulatory pipeline and for those that do, there is little follow-up post-registration. In addition, antimalarial drug prices vary widely within the same drug class. During the era of ACTs, there is a need to ensure that these new drugs are registered and monitored post-registration to ensure their continued safety, quality and efficacy. Regulatory mechanisms and price controls must be strengthened and enforced to improve the use of drugs in the retail sector. In addition, nation-wide, high profile communication strategies should accompany drug policy changes to bring demand for antimalarial drugs in line with policy.

## Authors' contributions

AAA was responsible for the study conception and design, data analysis, interpretation and writing of the manuscript. RWS supervised the studies overall and contributed substantially to redrafting the manuscript.
